# Serological Response to BNT162b2 Anti-SARS-CoV-2 Vaccination in Patients with Inflammatory Rheumatic Diseases: Results From the RHEUVAX Cohort

**DOI:** 10.3389/fimmu.2022.901055

**Published:** 2022-06-17

**Authors:** Daniele Mauro, Antonio Ciancio, Claudio Di Vico, Luana Passariello, Gelsomina Rozza, Maria Dora Pasquale, Ilenia Pantano, Carlo Cannistrà, Laura Bucci, Silvia Scriffignano, Flavia Riccio, Martina Patrone, Giuseppe Scalise, Piero Ruscitti, Maria Vittoria Montemurro, Antonio Giordano, Maria Teresa Vietri, Francesco Ciccia

**Affiliations:** ^1^ Rheumatology Unit, Department of Precision Medicine, Università degli Studi della Campania “L.Vanvitelli”, Naples, Italy; ^2^ Unit of Clinical and Molecular Pathology, Department of Precision Medicine, Università degli Studi della Campania “L.Vanvitelli”, Naples, Italy; ^3^ Rheumatology Unit, Department of Biotechnological & Applied Clinical Sciences, University of L’Aquila, L’Aquila, Italy; ^4^ Clinical Directorate, University Hospital of Università degli Studi della Campania “L. Vanvitelli”, Naples, Italy; ^5^ Head Office, University Hospital of Università degli Studi della Campania “L. Vanvitelli”, Naples, Italy

**Keywords:** COVID-19, vaccines, autoimmunity, rheumatic and muscoluskeletal disease, arthritis, connective tissue disease (CTD)

## Abstract

**Objective:**

In the light of the current COVID-19 epidemic and the availability of effective vaccines, this study aims to identify factors associated with non-response to anti-SARS-CoV-2 vaccines as immunological alteration associated with immune rheumatic diseases (IRD) and immunosuppressive medications may impair the response to vaccination.

**Methods:**

Volunteers in the health profession community with IRD, age, and sex-matched controls (CTRL) who underwent vaccination with two doses of BNT162b2 were recruited for this study. Anti-Trimeric Spike protein antibodies were assayed eight ± one weeks after the second vaccine dose. Univariate and logistic regression analyses were performed to identify factors independently associated with non-response and low antibody titers.

**Results:**

Samples were obtained from 237 IRD patients (m/f 73/164, mean age 57, CI 95% [56-59]): 4 autoinflammatory diseases (AI), 62 connective tissue diseases (CTD), 86 rheumatoid arthritis (RA), 71 spondylarthritis (SpA) and 14 vasculitis (Vsc). 232 CTRL were recruited (m/f 71/161, mean age 57, CI 95% [56-58]). Globally, IRD had a lower seroconversion rate (88.6% vs 99.6%, CI 95% OR [1.61-5.73], p<0.001) and lower antibody titer compared to controls (median (IQR) 403 (131.5-1012) versus 1160 (702.5-1675), p<0.001). After logistic regression, age, corticosteroid (CCS), Abatacept and Mycophenolate Mofetil (MMF) use were associated with non-response. Lower antibody titer was associated with the use of MMF, ABA, CCS, Rituximab, tumor necrosis factor inhibitor, JAK inhibitors, and higher age.

**Conclusion:**

The response to anti-SARS-CoV-2 vaccines is often impaired in IRD patients under treatment and may pose them at higher risk of severe COVID-19. Specific vaccination protocols are desirable for these patients.

## Introduction

It is widely accepted that patients with autoimmune inflammatory rheumatic disease are at higher risk of infection, partially due to treatment (1). In the initial phases of the SARS-CoV-2 pandemic, patients affected by autoimmune and inflammatory rheumatic diseases (IRD) raised concerns about their potentialy higher risk of getting infected and developing severe COVID-19. Several controversial data were made available during the last two years (2). To date, globally considered, the literature suggests that IRD are at higher risk of infection and burdened by higher mortality due to COVID-19 than the general population (3).

Comorbidities associated with higher risk for severe COVID-19 and death are frequent in some IRD patients, such as inflammatory lung disease, cardiovascular disease, and chronic kidney disease. Although not particularly strong at population levels, the risk for a worse prognosis can be exceptionally high in some patients (2, 4).

Immediately after the publication of the trials demonstrating the efficacy of the SARS-CoV-2 vaccine in mounting immunity against SARS-CoV-2 and preventing a severe form of COVID-19 and death, the need for prioritization of IRD patients to shield them from SARS-CoV-2 infection appeared evident. Rheumatologists and the scientific community have developed a strong awareness of the importance of vaccination in IRD patients and recommendations regarding the type of vaccines to be used and the timing concerning disease activity and treatment are available.

Vaccination against SARS-CoV-2 was demonstrated to be effective in IRD patient, by reducing the mortality and risk of hospitalization (5). However, a large body of evidence is accumulating on the impact of rheumatic disease and immunosuppressive treatment in response to vaccination, demonstrating a reduction in the immune response for some medication or suggesting a reduction in the titer for others (2).

While evidence from systematic reviews on other vaccines shows some impact of corticosteroids and csDMARDs, such as Methotrexate (MTX), on vaccine immunogenicity, data regarding some bDMARDs and small molecules, such as abatacept (ABA) and JAK inhibitors (JAKi), are now available but limited (6–8).

The European Alliance of Association for Rheumatology (EULAR) task force was of the opinion that the data on anti-CD20 therapy are most compelling, followed by data on mycophenolate mofetil (MMF) and glucocorticoids (9). Data on methotrexate, JAKi, and abatacept were judged not yet consistent/robust (9). Meanwhile, more data for ABA and JAKi are coming to light from studies with sample sizes ranging to 11 to 54 patients demonstrating reduction in antibody titers (10–13).

In order to contribute to the current knowledge regarding the impact of IRD and treatment on the response to SARS-CoV-2, we present here the result of an observational cross-sectional study evaluating the serological response and the persistence of antibodies at eight weeks in IRD patient cohort and non-IRD patient controls.

## Materials and Methods

### Patient and Control Population Recruitment

In line with the local recommendations, patients affected by IRD in follow-up in the Unit of Rheumatology of the University Hospital “L. Vanvitelli” were invited to receive the vaccination within the same center. From April 1, 2021 to June 30, 2021, 699 IRD outpatients received two doses of the BNT162b2 mRNA anti-SARS-CoV-2 vaccine, three weeks apart. After the approval of an internal ethics board, the possibility to perform a serological test to quantify the antibody response against SARS-CoV-2 was offered to patients eight weeks later. Informed consent was acquired, and serum and clinical data were collected. A population of sex- and age-matched healthy controls belonging to the hospital’s clinical and nonclinical personnel was used as control. Inclusion criteria were having a diagnosis of rheumatic disease and having received BNT162b2 vaccine 8 weeks before the sample collection. For the controls, only individuals with a diagnosis of rheumatic disease, immunodeficiency or under immunosuppressive treatment were excluded. At the time of sample collection, history of past-COVID-19, pharmacologic history, cancer history, and information regarding the disease duration were collected. Patients and the public were not involved in this research’s design, reporting, and dissemination.

### Anti-SARS-CoV-2 Testing

The collected sera from both patients and controls were analyzed in batch after being stored at -80°C using Liaison^®^ SARS-CoV-2 TrimericS IgG CLIA kits provided by DiaSorin^®^ following manufacturer protocol. The levels of anti-SARS-CoV-2 IgG antibodies were expressed in World Health Organization International Standard (NIBSC code. 20/268) binding arbitrary unit (BAU/ml). Samples with values of ≥33.8 BAU/ml were considered positive. Patients and the public were not involved in this research’s design, reporting, and dissemination.

### Statistical Analysis

Depending on their distribution, variables are reported as medians and IQRs or means and standard deviation (SD). Participants were stratified into responders and non-responders according to Binding Antibody Unit/mL level using 33.8 BAU/mL as the cut-off. For the analysis, the antibody titer was log-transformed to improve normality. For categorical variables, including seroconversion rate, the differences in frequency were tested using χ2 and Fisher’s exact test. Factors possibly associated with seroconversion were then tested in a binary logistic model using backward stepwise selection (p cut-off for elimination 0.2).

Differences in titer were tested by Mann-Whitney-U test for two-group comparison or Kruskal-Wallis test for three or more comparisons. The effect of demographic, diagnosis, and treatment of the antibody titers was further tested in a linear regression model using backward stepwise selection (p-value cut-off for elimination 0.2)

Statistical analysis and graphical presentation of the data were performed using GraphPad Prism (V.9.1.0), IBM SPSS Statistics (V.26), and RStudio (V. 2021.9.1.372) were used for the statistics and graphical presentation of the data.

## Results

### RHEUVAX Cohort

Two hundred thirty-seven patients were enrolled and included in the study: 71 with SpA (spondylarthritis), 62 with CTD (connective tissue diseases) including systemic sclerosis, systemic lupus erythematosus, Sjogren’s syndrome, 86 with RA (rheumatoid arthritis), 14 with Vsc (vasculitides) and 4 with AI (autoinflammatory diseases). The mean age was 57 CI 95% [56-59] with an M: F ratio of 73:164. 64.9% received csDMARDs and 49.8% were under treatment with either a bDMARD or a tsDMARDs. Out of 237 patients, 13 (5.5%) had an anamnesis positive for past Sars-CoV-2 infection confirmed serologically, by PCR or Antigenic test.

The demographic characteristics are shown in **Table 1**. The sex and age-matched control cohort was composed of 232 individuals, mean age of 57 CI 95% [56-58] with an M: F ratio of 71:161.

**Table 1:** Demographic characteristics of patients and controls.

	IRD	CTRL
**n**	*237*	*232*
**Age** mean [CI 95%]	*57 (56-59)*	*57 [56-58]*
**M:F**	*73/164*	*71/161*
**Diagnosis of IRD n (%)**	*237 (100)*	*0 (0)*
**RA n (%)**	*86 (36.3)*	*-*
**SpA n (%)**	*71 (30.0)*	*-*
- axSpA	*14 (5.9)*	*-*
- PsA	*48 (20.3)*	*-*
- Other	*9 (3.8)*	*-*
**CTD n (%)**	*62 (26.2)*	*-*
- UCTD	*7 (2.9)*	*-*
- SSc	*21 (8.9)*	*-*
- SLE	*20 (8.4)*	*-*
- pSS	*3 (1.3)*	*-*
- IM	*6 (2.5)*	*-*
- MCTD	*1 (0.4)*	*-*
- APS	*4 (1.7)*	*-*
**Vsc n (%)**	14 (5.9)	–
**AI n (%)**	*4 (1.7)*	*-*
**Treatment**		–
**csDMARD n (%)**	154(64.9)	–
- MTX	94 (39.7)	–
- HCQ	14 (5.9)	–
- MMF	16 (6.8)	–
- AZA	*16 (6.8)*	*-*
- SSZ	*9 (3.8)*	*-*
- LEF	*2 (0.8)*	*-*
- Colchicine	*3 (1.3)*	*-*
**bDMARD and tsDMARD n (%)**	*118 (49.8)*	*-*
- TNFi	*49 (20.7)*	*-*
- ABA	*15 (6.3)*	*-*
- UST	*7 (3.0)*	*-*
- IL-17i	*14 (5.9)*	*-*
- IL-6i	*12 (5.0)*	*-*
- IL-1i	*2 (0.8)*	*-*
- RTX	*4 (1.7)*	*-*
- BEL	*1 (0.4)*	*-*
- PDE4i	*1 (0.4)*	*-*
- JAKi	*18 (7.6)*	*-*

IRD, Immune rheumatic disease; IQR, Interquartile range; RA, Rheumatoid Arthritis; SpA, Spondyloarthritis; axSpA, axial Spondyloarthritis; PsA, Psoriatic Arthritis; CTD, Connective tissue diseases; UCTD, Undifferentiated Connective Tissue Disease; SSc, Systemic Sclerosis; SLE, Systemic Lupus Erythematosus; pSS, primary Sjögren Syndrome; IM, Inflammatory Myositis; MCTD, Mixed Connective Tissue Disease; APS, Antiphospholipid Syndrome; Vsc, Vasculitides; AI, Autoinflammatory Diseases; csDMARD, conventional synthetic Disease-Modifying Antirheumatic Drug; MTX, Methotrexate; HCQ, Hydroxychloroquine; MMF, Mycophenolate Mofetil; AZA, Azathioprine; SSZ, sulfasalazine; LEF, Leflunomide; bDMARD, biological Disease-Modifying Antirheumatic Drug; tsDMARD, targeted synthetic Disease-Modifying Antirheumatic Drug; TNFi, TNF alpha-inhibitors; ABA, Abatacept; UST, Ustekinumab; IL-17i, IL-17-inhibitors; IL-6i, IL-6-inhibitors; IL-1i, IL-1-inhibitors; RTX, Rituximab; BEL, belimumab; PDE4i, phosphodiesterase 4-inhibitors; JAKi, Janus kinase-inhibitors.

### Seroconversion Rates in IRD Patients Compared to Controls

**Figure 1:**
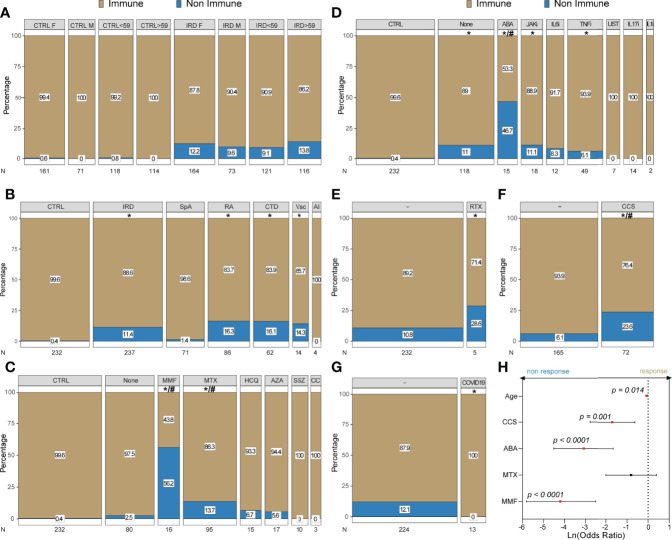
Seroconversion rates after BNT162B2 vaccine in IRD and controls: Mosaic plots represent the percentage of serological response and non-response in controls (CTRL) and patients (IRD); bar size is proportional with the sample size (n) of the group. **(A)** stratification according to sex and age (> vs < the median); **(B)** percentage of response across different IRD diagnoses; **(C)** response in patients treated with csDMARDs; **(D)** response in patients treated with bDMARDs or tsDMARDs; In **(E–G)** stratification performed according to RTX treatment in the last 12 months, CCS use and past Sars-Cov-2 infection, respectively. In **(H)** forest plot showing factors associated with of seroconversion identified by binary stepwise logistic regression displayed as 95% CI of Ln(Odds Radio) and p-value. Full analysis results and p-values are reported in **Supplementary Table 1**. *: statistically significant vs control population; #: statistically significant vs not taking csDMARDs or bDMARDs/tsDMARDs; */# = p < 0.05.

In the control population, 99.6% of subjects achieved seroconversion while 88.6% of patients with IRD showed above-threshold anti-spike protein antibody titer, 95% CI of Ln (OR) of non-response [1.61-5.73], p < 0.001. The difference was also maintained after stratification by age and sex, Conversely, sex and age did not influence the seroconversion rate within groups (**Figure 1A**).

Specifically, univariate analysis showed reduced seroconversion rates compared to CTRL in CTD, RA and Vcs (83.9%, 83.7% and 85.7%, respectively), but not in AI and SpA (100% and 98.6%), **Figure 1B**.

#### Impact of Treatment on Seroconversion

Similarly, both treatment with csDMARDs and bDMARDs/tsDMARDs were associated with a lower seroconversion rate compared to controls [percentage (Ln (OR) of non-response) 84.0% (2.09-6.12) and 88.24 (1.60-5.79), respectively p < 0.001] as shown in Figure. Specifically, the ratio of seroconversion was lower in mycophenolate mofetil (MMF), methotrexate (MTX), abatacept (ABA), Janus Kinase inhibitors (JAKi) and tumor necrosis factor inhibitors (TNFi), as shown in **Figures 1C, D**. Similarly, when the rate in patients who received Rituximab (RTX) in the past 12 months was compared to control, a significantly lower rate of responders was observed (99.6% vs 71.4%, [1.54-5.57], p < 0.01) however, this difference was not statistically significant when the level was compared to the other IRD patients not treated with RTX (**Figure 1E**). Independent of the doses, corticosteroid therapy (CCS) was associated with a higher rate of non-response and only 76.9% of patients developed antibodies [Ln (OR) of non-response (2.46-6.62), and this proportion was smaller than CTRL and IRD groups not taking CCS (93.9%), p < 0.001] (**Figure 1F**).

#### Seroconversion in IRD Patients With Past Sars-CoV-2 Infection

Finally, out of 237 patients, only 13 had a documented past Sars-CoV-2 infection and all the patients maintained/developed a serological response after the vaccination (**Figure 1G**). Detailed response percentage and comparison are shown in **Supplementary Table 1**

#### Binary Logistic Regression Model

In order to account for confounding variables, a binary logistic regression model was implemented using the data of the 224 IRD patients who had no evidence of prior infection to investigate associations with antibody positivity. After backward stepwise regression only, MMF, ABA, CCS and age remained significant associated factors (at p<0.05 level) of serological response (**Figure 1H**). All but MTX were negatively associated with the seroconversion, corrected ln (OR) 95% CI 0.03-0.08 for MMF, 0.01-0.19 for ABA, 0.06-0.54 for CCS, and 0.89-0.99 for age. Suggesting that treatment with MMF or ABA was more strongly associated with non-response to Sars-CoV-2 vaccination (**Figure 1H**).

### Antibody Titers

**Figure 2:**
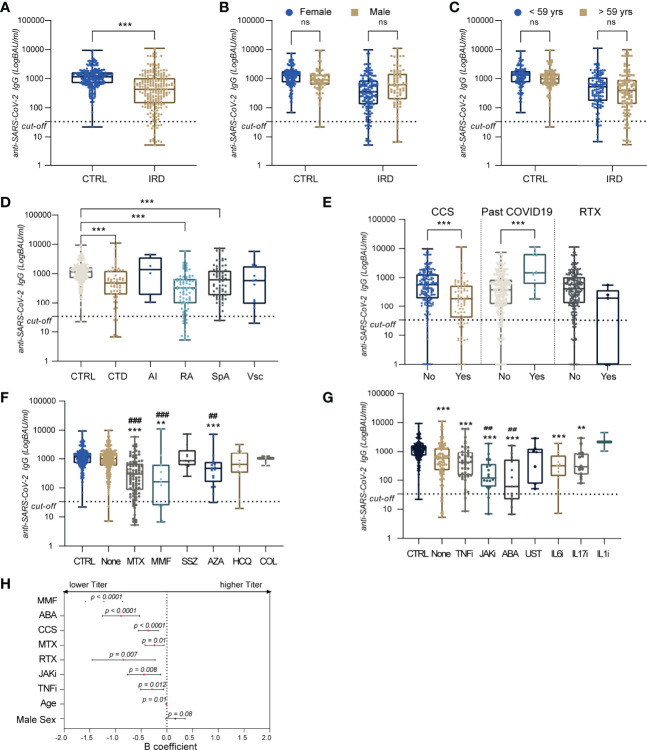
Antibody titers after BNT162B2 vaccine in IRD and controls:Box and whiskers plot with individual data point anti-Sars-CoV-2 antibody tier expressed in BAU/mL, the box represents the IQR, line the median and hinges extend from the minimum to maximum value **(A–G)**. In **(A)** comparison of titer between control (CTRL) and patients (IRD). In **(B, C)** titer after stratification for sex and age (> vs < the median), respectively. Levels of anti-Sars-Cov2 antibodies in the different patient’s groups are reported in **(D)**. In **(E)** antibody levels after stratification for CCS use, past COVID19 and Rituximab treatment in the last 12 months. Antibody levels are compared across different csDMARDs **(F)** and bDMARDs/tsDMARDs **(G)** use. In **(H)** forest plot showing factors associated with seroconversion identified by linear stepwise regression displayed as 95% CI of B and p-value. *: statistically significant vs control population; #: statistically significant vs not taking csDMARDs or bDMARDs/tsDMARDs; **/## = p < 0.01; ***/### = p < 0.001. ns, not significant.

When analyzing antibody titers after vaccination, we found that IRD patients showed overall lower mean titers compared to controls (**Figure 2A**): [median (IQR) 403 (131.5-1012) vs 1160 (702.5-1675), p<0.001].

No differences were identified when patients and controls were stratified by age, group, and sex (**Figures 2B–C**). When we looked at the different IRD diseases, CTD, RA, and SpA had lower titer levels p <0.001: median (IQR) 415 (132-1233). 314 (61-670) and 622 (184-1280), respectively (**Figure 2D**). Past COVID-19 was associated with higher titers than patients without prior infection (**Figure 2D**). CCS users surprisingly showed lower titer compared to patients not under CCS (IQR) 189 (39.7-534.5) vs. 576 (192-1300), p < 0.001; likely due to small sample size (n = 5), and no differences were observed when studying the titer of patients who received RTX in last 12 months (**Figure 2E**).

Nearly all treatments appeared to influence antibody titers, except for Sulfasalazine, Hydroxychloroquine (HCQ), Colchicine (COL), IL1 inhibitors (IL1i) and Ustekinumab (UST). Treatment with Azathioprine (AZA), MTX, and MMF led to lower titer compared either to controls and other patients not taking any csDMARDs (**Figure 2F**): median BAU/mL (IQR) 466 (167.5-783.5) for AZA, 276 (65.5-673) for MTX and 25 (0-318.5) for MMF.

Similarly, the levels of anti-Sars-Cov-2 antibodies were investigated in bDMARDs and tsDMARDs treated patients observing a significant reduction compared to the controls for all the drugs apart from IL1i (**Figure 2G**): median BAU/mL (IQR) 34.5 (11.7-435) for ABA, 314 (141.3-736.3) for IL6i, 119.5 (59.60-374.5) for JAKi, 397 (142.5-948) for TNFi. Patients under treatment with ABA and JAKi had the lowest titer values and these were significantly lower than IRD patients not treated with any bDMARDs or tsDMARDS, p < 0.01.

#### Linear Regression Model

After performing a backward stepwise linear regression model using anti-Sars-Cov-2 titer as the independent variable and including all treatment data, Rituximab, patient age, and sex, we identified that MMF, ABA, CCS, MTX, JAKi, TNFi, age and male sex was associated with antibody titer (**Figure 2H**). Specifically, increasing age was negatively associated with the titer (B 95% CI [-0.02;0.005]) and the treatments: B 95% CI [-1.58;-0.86] for MMF, [-4.42;-0.06] for MTX, [-1.25;-0.53] for ABA, [-0.77;-0.12] for JAKi, [-0.51;-0.06] for TNFi, [-1.46;-0.23] for RTX and [-0.56;-0.16] for CCS. This confirmed that MMF and ABA use were the strongest influencer antibodies levels (**Figure 2H**).

## Discussion

Randomized clinical trials and real-life data demonstrated the safety and efficacy of the currently available mRNA vaccine leading to approval nearly worldwide (14, 15). The immunological response is commonly altered in IRD patients and it is the target of most of the current treatments (16).

Independent studies following the approval of mRNA vaccines demonstrated their efficacy and safety also in IRD and immunocompromised patients (6). However, a higher percentage of non-responders are observed in immunocompromised patients. A sizable proportion of IRD patients fails to mount a serological response against Sars-Cov-2: in our cohort globally 11.4% had no detectable levels of IgG anti-Sars-Cov-2 with a peak of 56.2% in MMF treated patients, which is in line with other studies (11). Therefore, there is an emerging and urgent need to identify vulnerable, unprotected IRD patients and elaborate strategies aiming to maximize the protection against severe COVID-19 in these patients. This work aimed to identify the cause of the lack of antibody-mediated immune response against SARS-CoV-2 in a cohort of Italian adult IRD patients. To date, this study is among the largest controlled cohorts evaluating the BNT162b2 vaccine in a rheumatologic setting, thus adding a significant contribution to previous similar studies.

As evidenced in our cohort, although the BNT162b2 vaccine showed a good immunogenicity profile according to seroconversion rate, a considerable portion of patients affected by IRD failed to mount an appropriate humoral response. This data seems consistent with evidence shown in other cohorts, with comparatively similar seroconversion rates (11, 17, 18).

As expected, antibody titers appeared to be reduced in IRD patients compared to controls. Nearly all disease categories were affected by this reduction, except for patients with autoinflammatory diseases.

Treatment with CCS, MMF, and ABA were consistently associated with failed seroconversion and lower anti-spike antibody titers, thus suggesting that these medications alone might be responsible for ineffective vaccination.

In the light of the common use of MMF in rheumatology to treat severe systemic conditions, including inflammatory lung disease and glomerulonephritis that are per se associated with a higher risk of death due to COVID-19, this observation appears of high clinical relevance (4, 19).

Such associations find confirmation in other studies on anti-SARS-CoV-2 vaccines (11, 17, 20, 21) and mirror what we know from other vaccination data (22–26).. At the same time, MMF negatively impacted immunogenicity after the HPV vaccine in patients with Systemic Lupus Erythematosus (27) and response to influenza vaccine in kidney transplant recipients (28). In this regard, the evidence that MMF impairs COVID-19 vaccine immune response is widely recognized; and conversely, data on ABA were still considered by some authors and by the EULAR as inconclusive for the risk of bias and the small sample size of the published study (9). However, more data are accumulating (11, 13) and the results of this controlled study gains added value as it contributes to the identification of ABA as a negative predictor of response.

Corticosteroids appeared to have either a relatively neutral (29) or a moderately negative influence on seroconversion rates after vaccination against influenza viruses (30, 31). Using regression analysis, we tried to pull apart the contribution of CCS, demonstrating a negative effect on both seroconversion rate and antibody titer. One of the limitations of our study is the stratification of patients in CCS users versus non-CCS users, not taking into consideration the prednisone equivalent dose in the analysis. This choice was driven by the need for an adequate sample size in each group and allowed CCS identification as a factor associated with lower antibody production. The effect size will likely be even more prominent in patients taking high doses of corticosteroids.

An unusual finding was the relatively modest effect of Rituximab on seroconversion rates in our cohort. As evidenced in other studies on COVID-19 vaccines and data from studies on influenza and pneumococcal vaccines, Rituximab exerts a potent action on antibody production and is known to compromise immunogenicity of COVID-19 vaccine, as well as other vaccines (6, 32–34). Furthermore, B cell depleting therapy seems to influence also the T cells function by compromising the circulating T follicular helper cell response and augmenting the CD8 T cell induction (35).

However, the patients treated with RTX in the last 12 months were only five in our cohort.

The negative effect exerted by MMF, ABA, CCS, MTX, JAKi, RTX, and TNFi is partially in line with previously reported information (2, 7, 21, 36–38) and it is of relevance independent from the definition of seroconversion. Although a clear cut-off of antibody titer associated with protection is lacking, the data available demonstrates that the protection drops during subsequent months (39) and this supports the policy of administering a third and fourth booster dose (40–42) and maybe more in the future. Immunosuppressive therapy may lead to a faster loss of protection in IRD patients. In this context, the timing of testing chosen in this study may be more informative. One limitation of the study is the lack of longitudinal data; however, testing two months (8 weeks) after the second dose may allow catching a steady state.

One criticism against serological studies is the lack of information about cell-mediated immunity, particularly T-cell response and to the neutralization of the emerging variants, including the Omicron. Since T-cell immunity may confer protection also in the absence of antibodies (43–45), antibody titer may be only the tip of the iceberg (46–49). Most commonly, drugs such as ABA and MMF impair both T cells and B cells’ (50) mediated response, possibly exposing the patients to a higher susceptibility to severe COVID-19. A low serological response may well indicate a poor response and protection in these cases.

At the moment, the accumulating evidence on the impact of these treatments on vaccine immunogenicity should prompt a reflection about whether it would be advisable for patients to temporarily stop therapy with MMF and ABA and at least reduce daily corticosteroid dose before vaccination. Indeed, data from a small cohort suggests that adopting a temporary hold policy for MMF was associated with higher anti-SARS-CoV-2 antibody titers compared to patients who did not withhold the medication (92% vs 65% seroconversion rate (51). However, this strategy is still not backed up by solid evidence and further studies are required to establish an exact timing for the temporary hold of each treatment to achieve an adequate balance between vaccine efficacy and disease control.

In this context, clinical guidance and recommendations on the COVID-19 vaccination for patients with rheumatic and musculoskeletal disease has been released and recently updated by the American College of Rheumatology (ACR) and the European Alliance of Associations for Rheumatology (EULAR) (9, 52).

The decision to whether temporarily withhold a treatment for IRD is particularly delicate and requires reliable scientific data to perform a risk-benefit evaluation. The temporary interruption of the treatment may expose the patients to a higher risk of flare and needs to be balanced with the risk of severe COVID-19.

Finally, a universally accepted cut-off for the serological antibody titer offering protection to severe COVID-19 has not been identified, however it appears relevant that a proportion of patients did not have any serological response, and these are likely to be at higher risk. Seroconversion rates among patients with autoimmune and inflammatory rheumatic disease are lower overall compared to controls, and this effect is imputable to concomitant therapy.

Treatment with corticosteroids, MMF, ABA, and older age are associated with the absence of serological response to the BNT162b2 mRNA anti-SARS-CoV-2 vaccine. Treatment with MMF, ABA, CCS, MTX, JAKi, TNFi, and RTX reduces the antibody titer and may accelerate the drop in immunity against SARS-CoV-2.

Shared evidence-based strategies (51) should be developed to identify patients at high risk of being unprotected and improve their response to SARS-CoV-2 vaccines.

## Data Availability Statement

The raw data supporting the conclusions of this article will be made available by the authors, without undue reservation.

## Ethics Statement

The studies involving human participants were reviewed and approved by Ethical Committee of the University of Campania L. Vanvitelli. The patients/participants provided their written informed consent to participate in this study.

## Author Contributions

DM, AC, CDV, and FC developed the study protocol. DM, AC, CDV, GR, MDP, CC, FR, SS, MP, and LB contributed to enrollment, sample collection, data collection and data quality control. MV and LP performed serological analysis. DM performed data analysis and interpretation and prepared the figures. DM and AC drafted the manuscript. All the authors contributed to interpreting the data and revised the manuscript critically for important intellectual content.

## Conflict of Interest

The authors declare that the research was conducted in the absence of any commercial or financial relationships that could be construed as a potential conflict of interest.

## Publisher’s Note

All claims expressed in this article are solely those of the authors and do not necessarily represent those of their affiliated organizations, or those of the publisher, the editors and the reviewers. Any product that may be evaluated in this article, or claim that may be made by its manufacturer, is not guaranteed or endorsed by the publisher.

## References

[1] Falagas ME Manta KG Betsi GI Pappas G. Infection-Related Morbidity and Mortality in Patients With Connective Tissue Diseases: A Systematic Review. Clin Rheumatol (2007) 26:663–70. doi: 10.1007/S10067-006-0441-9 17186117

[2] Kroon FPB Najm A Alunno A Schoones JW Landewé RBM Machado PM. Risk and Prognosis of SARS-CoV-2 Infection and Vaccination Against SARS-CoV-2 in Rheumatic and Musculoskeletal Diseases: A Systematic Literature Review to Inform EULAR Recommendations. Ann Rheum Dis (2022) 81(3):422–32. doi: 10.1136/annrheumdis-2021-221575 34876462

[3] Conway R Grimshaw AA Konig MF Putman M Duarte-García A Tseng LY. SARS-CoV -2 Infection and COVID -19 Outcomes in Rheumatic Disease: A Systematic Literature Review And Meta-Analysis. Arthritis Rheumatol (2022) 74(5):766–75. doi: 10.1002/art.42030 PMC9011807 34807517

[4] Drake TM Docherty AB Harrison EM Quint JK Adamali H Agnew S. Outcome of Hospitalization for COVID-19 in Patients With Interstitial Lung Disease. An International Multicenter Study. Am J Respir Crit Care Med (2020) 202:1656–65. doi: 10.1164/RCCM.202007-2794OC PMC7737581 33007173

[5] Papagoras C Fragoulis GE Zioga N Simopoulou T Deftereou K Kalavri E. Better Outcomes of COVID-19 in Vaccinated Compared to Unvaccinated Patients With Systemic Rheumatic Diseases. Ann Rheum Dis (2021) 81(7):annrheumdis-2021-221539. doi: 10.1136/annrheumdis-2021-221539 34758975

[6] Rondaan C Furer V Heijstek MW Agmon-Levin N Bijl M Breedveld FC. Efficacy, Immunogenicity and Safety of Vaccination in Adult Patients With Autoimmune Inflammatory Rheumatic Diseases: A Systematic Literature Review for the 2019 Update of EULAR Recommendations. RMD Open (2019) 5:e001035. doi: 10.1136/rmdopen-2019-001035 31565247 PMC6744079

[7] Winthrop KL Silverfield J Racewicz A Neal J Lee EB Hrycaj P. The Effect of Tofacitinib on Pneumococcal and Influenza Vaccine Responses in Rheumatoid Arthritis. Ann Rheum Dis (2016) 75:687–95. doi: 10.1136/annrheumdis-2014-207191 PMC4819610 25795907

[8] Winthrop KL Bingham CO Komocsar WJ Bradley J Issa M Klar R. Evaluation of Pneumococcal and Tetanus Vaccine Responses in Patients With Rheumatoid Arthritis Receiving Baricitinib: Results From a Long-Term Extension Trial Substudy. Arthritis Res Ther (2019) 21:102. doi: 10.1186/s13075-019-1883-1 30999933 PMC6471863

[9] Landewé RBM Kroon FPB Alunno A Najm A Bijlsma JW Burmester G-RR. EULAR Recommendations for the Management and Vaccination of People With Rheumatic and Musculoskeletal Diseases in the Context of SARS-CoV-2: The November 2021 Update. Ann Rheum Dis (2022) annrheumdis-2021-222006. doi: 10.1136/annrheumdis-2021-222006 35197264

[10] Deepak P Kim W Paley MA Yang M Carvidi AB Demissie EG. Effect of Immunosuppression on the Immunogenicity of mRNA Vaccines to SARS-CoV-2: A Prospective Cohort Study. Ann Intern Med (2021) 174:1572–85. doi: 10.7326/M21-1757 PMC8407518 34461029

[11] Furer V Eviatar T Zisman D Peleg H Paran D Levartovsky D. Immunogenicity and Safety of the BNT162b2 mRNA COVID-19 Vaccine in Adult Patients With Autoimmune Inflammatory Rheumatic Diseases and in the General Population: A Multicentre Study. Ann Rheum Dis (2021) 80:1330–8. doi: 10.1136/annrheumdis-2021-220647 34127481

[12] Wieske L van Dam KPJ Steenhuis M Stalman EW Kummer LYL van Kempen ZLE. Humoral Responses After Second and Third SARS-CoV-2 Vaccination in Patients With Immune-Mediated Inflammatory Disorders on Immunosuppressants: A Cohort Study. Lancet Rheumatol (2022) 4(5):e338-50. doi: 10.1016/S2665-9913(22)00034-0 35317410 PMC8930018

[13] Medeiros-Ribeiro AC Bonfiglioli KR Domiciano DS Shimabuco AY da Silva HC Saad CGS. Distinct Impact of DMARD Combination and Monotherapy in Immunogenicity of an Inactivated SARS-CoV-2 Vaccine in Rheumatoid Arthritis. Ann Rheum Dis (2022) 81:710–9. doi: 10.1136/annrheumdis-2021-221735 35135832

[14] Dagan N Barda N Kepten E Miron O Perchik S Katz MA. BNT162b2 mRNA Covid-19 Vaccine in a Nationwide Mass Vaccination Setting. N Engl J Med (2021) 384:1412–23. doi: 10.1056/NEJMOA2101765 PMC7944975 33626250

[15] Thomas SJ Moreira ED Kitchin N Absalon J Gurtman A Lockhart S. Safety and Efficacy of the BNT162b2 mRNA Covid-19 Vaccine Through 6 Months. N Engl J Med (2021) 385:1761–73. doi: 10.1056/NEJMOA2110345 PMC8461570 34525277

[16] Mehta B Pedro S Ozen G Kalil A Wolfe F Mikuls T. Serious Infection Risk in Rheumatoid Arthritis Compared With Non-Inflammatory Rheumatic and Musculoskeletal Diseases: A US National Cohort Study. RMD Open (2019) 5:e000935. doi: 10.1136/rmdopen-2019-000935 31245055 PMC6560658

[17] Braun-Moscovici Y Kaplan M Braun M Markovits D Giryes S Toledano K. Disease Activity and Humoral Response in Patients With Inflammatory Rheumatic Diseases After Two Doses of the Pfizer mRNA Vaccine Against SARS-CoV-2. Ann Rheum Dis (2021) 80:1317–21. doi: 10.1136/annrheumdis-2021-220503 34144967

[18] Simon D Tascilar K Fagni F Krönke G Kleyer A Meder C. SARS-CoV-2 Vaccination Responses in Untreated, Conventionally Treated and Anticytokine-Treated Patients With Immune-Mediated Inflammatory Diseases. Ann Rheum Dis (2021) 80:1312–6. doi: 10.1136/annrheumdis-2021-220461 PMC8103562 33958324

[19] Sakthiswary R Chuah HY Chiang KS Liew YS Muhammad Aizat NA. COVID-19 in Systemic Lupus Erythematosus: A Pooled Analysis and Systematic Review of Case Reports and Series. Lupus (2021) 30:1946–54. doi: 10.1177/09612033211045057 34565208

[20] Picchianti-Diamanti A Aiello A Laganà B Agrati C Castilletti C Meschi S. ImmunosuppressiveTherapies Differently Modulate Humoral- and T-Cell-Specific Responses to COVID-19 mRNA Vaccine in Rheumatoid Arthritis Patients. Front Immunol (2021) 12:740249. doi: 10.3389/fimmu.2021.740249 34594343 PMC8477040

[21] Ruddy JA Connolly CM Boyarsky BJ Werbel WA Christopher-Stine L Garonzik-Wang J. High Antibody Response to Two-Dose SARS-CoV-2 Messenger RNA Vaccination in Patients With Rheumatic and Musculoskeletal Diseases. Ann Rheum Dis (2021) 80:1351–2. doi: 10.1136/annrheumdis-2021-220656 PMC8843949 34031032

[22] Ribeiro AC Laurindo IM Guedes LK Saad CG Moraes JC Silva CA. Abatacept and Reduced Immune Response to Pandemic 2009 Influenza A/H1N1 Vaccination in Patients With Rheumatoid Arthritis. Arthritis Care Res (Hoboken) (2013) 65:476–80. doi: 10.1002/acr.21838 22949223

[23] Adler S Krivine A Weix J Rozenberg F Launay O Huesler J. Protective Effect of A/H1N1 Vaccination in Immune-Mediated Disease–a Prospectively Controlled Vaccination Study. Rheumatology (2012) 51:695–700. doi: 10.1093/rheumatology/ker389 22171015

[24] Alten R Bingham CO Cohen SB Curtis JR Kelly S Wong D. Antibody Response to Pneumococcal and Influenza Vaccination in Patients With Rheumatoid Arthritis Receiving Abatacept. BMC Musculoskelet Disord (2016) 17:231. doi: 10.1186/s12891-016-1082-z 27229685 PMC4880815

[25] Crnkic Kapetanovic M Saxne T Jönsson G Truedsson L Geborek P. Rituximab and Abatacept But Not Tocilizumab Impair Antibody Response to Pneumococcal Conjugate Vaccine in Patients With Rheumatoid Arthritis. Arthritis Res Ther (2013) 15:R171. doi: 10.1186/ar4358 24286269 PMC3978887

[26] Migita K Akeda Y Akazawa M Tohma S Hirano F Ideguchi H. Effect of Abatacept on the Immunogenicity of 23-Valent Pneumococcal Polysaccharide Vaccination (PPSV23) in Rheumatoid Arthritis Patients. Arthritis Res Ther (2015) 17:357. doi: 10.1186/s13075-015-0863-3 26653668 PMC4675027

[27] Mok CC Ho LY Fong LS To CH. Immunogenicity and Safety of a Quadrivalent Human Papillomavirus Vaccine in Patients With Systemic Lupus Erythematosus: A Case–Control Study. Ann Rheum Dis (2013) 72:659–64. doi: 10.1136/annrheumdis-2012-201393 22589375

[28] Salles MJC Sens YAS Boas LSV Machado CM. Influenza Virus Vaccination in Kidney Transplant Recipients: Serum Antibody Response to Different Immunosuppressive Drugs. Clin Transplant (2010) 24:E17–23. doi: 10.1111/j.1399-0012.2009.01095.x 19758368

[29] Huang Y Wang H Tam WWS. Is Rheumatoid Arthritis Associated With Reduced Immunogenicity of the Influenza Vaccination? A Systematic Review and Meta-Analysis. Curr Med Res Opin (2017) 33:1901–8. doi: 10.1080/03007995.2017.1329140 28489423

[30] Crowe SR Merrill JT Vista ES Dedeke AB Thompson DM Stewart S. Influenza Vaccination Responses in Human Systemic Lupus Erythematosus: Impact of Clinical and Demographic Features. Arthritis Rheum (2011) 63:2396–406. doi: 10.1002/art.30388 PMC3149742 21598235

[31] Borba EF Saad CGS Pasoto SG Calich ALG Aikawa NE Ribeiro ACM. Influenza A/H1N1 Vaccination of Patients With SLE: Can Antimalarial Drugs Restore Diminished Response Under Immunosuppressive Therapy? Rheumatology (2012) 51:1061–9. doi: 10.1093/rheumatology/ker427 22298793

[32] Spiera R Jinich S Jannat-Khah D. Rituximab, But Not Other Antirheumatic Therapies, Is Associated With Impaired Serological Response to SARS- CoV-2 Vaccination in Patients With Rheumatic Diseases. Ann Rheum Dis (2021) 80:1357–9. doi: 10.1136/annrheumdis-2021-220604 33975857

[33] Ammitzbøll C Bartels LE Bøgh Andersen J Risbøl Vils S Elbæk Mistegård C Dahl Johannsen A. Impaired Antibody Response to the BNT162b2 Messenger RNA Coronavirus Disease 2019 Vaccine in Patients With Systemic Lupus Erythematosus and Rheumatoid Arthritis. ACR Open Rheumatol (2021) 3:622–8. doi: 10.1002/acr2.11299 PMC8426741 34273260

[34] Avouac J Miceli-Richard C Combier A Steelandt A Fogel O Mariaggi AA. Risk Factors of Impaired Humoral Response to COVID-19 Vaccination in Rituximab-Treated Patients. Rheumatology (2021) keab815. doi: 10.1093/rheumatology/keab815 PMC8689920 34726701

[35] Apostolidis SA Kakara M Painter MM Goel RR Mathew D Lenzi K. Cellular and Humoral Immune Responses Following SARS-CoV-2 mRNA Vaccination in Patients With Multiple Sclerosis on Anti-CD20 Therapy. Nat Med (2021) 27:1990–2001. doi: 10.1038/s41591-021-01507-2 34522051 PMC8604727

[36] Subesinghe S Bechman K Rutherford AI Goldblatt D Galloway JB. A Systematic Review and Metaanalysis of Antirheumatic Drugs and Vaccine Immunogenicity in Rheumatoid Arthritis. J Rheumatol (2018) 45:733–44. doi: 10.3899/jrheum.170710 29545454

[37] Migita K Akeda Y Akazawa M Tohma S Hirano F Ideguchi H. Pneumococcal Polysaccharide Vaccination in Rheumatoid Arthritis Patients Receiving Tacrolimus. Arthritis Res Ther (2015) 17:149. doi: 10.1186/s13075-015-0662-x 26036592 PMC4481124

[38] Franca ILA Ribeiro ACM Aikawa NE Saad CGS Moraes JCB Goldstein-Schainberg C. TNF Blockers Show Distinct Patterns of Immune Response to the Pandemic Influenza A H1N1 Vaccine in Inflammatory Arthritis Patients. Rheumatology (2012) 51:2091–8. doi: 10.1093/rheumatology/kes202 PMC7313849 22908326

[39] Cohn BA Cirillo PM Murphy CC Krigbaum NY Wallace AW. SARS-CoV-2 Vaccine Protection and Deaths Among US Veterans During 2021. Science (2021) 375(6578):331–6. doi: 10.1126/SCIENCE.ABM0620 PMC9836205 34735261

[40] Spitzer A Angel Y Marudi O Zeltser D Saiag E Goldshmidt H. Association of a Third Dose of BNT162b2 Vaccine With Incidence of SARS-CoV-2 Infection Among Health Care Workers in Israel. JAMA (2022) 327(4):341–9. doi: 10.1001/jama.2021.23641 PMC8749710 35006256

[41] Garcia-Beltran WF St. Denis KJ Hoelzemer A Lam EC Nitido AD Sheehan ML. mRNA-Based COVID-19 Vaccine Boosters Induce Neutralizing Immunity Against SARS-CoV-2 Omicron Variant. Cell (2022) 85(3):457–66.e4. doi: 10.1016/j.cell.2021.12.033 PMC8733787 34995482

[42] Stasi C Meoni B Voller F Silvestri C. SARS-CoV-2 Vaccination and the Bridge Between First and Fourth Dose: Where Are We? Vaccines (2022) 10:444. doi: 10.3390/vaccines10030444 35335075 PMC8953092

[43] Painter MM Mathew D Goel RR Apostolidis SA Pattekar A Kuthuru O. Rapid Induction of Antigen-Specific CD4+ T Cells Is Associated With Coordinated Humoral and Cellular Immunity to SARS-CoV-2 mRNA Vaccination. Immunity (2021) 54:2133–42.e3. doi: 10.1016/j.immuni.2021.08.001 34453880 PMC8361141

[44] Schwarzkopf S Krawczyk A Knop D Klump H Heinold A Heinemann FM. Cellular Immunity in COVID-19 Convalescents With PCR-Confirmed Infection But With Undetectable SARS-CoV-2–Specific IgG. Emerg Infect Dis (2021) 27:122–9. doi: 10.3201/eid2701.203772 33058753

[45] Prendecki M Clarke C Edwards H McIntyre S Mortimer P Gleeson S. Humoral and T-Cell Responses to SARS-CoV-2 Vaccination in Patients Receiving Immunosuppression. Ann Rheum Dis (2021) 80:1322–9. doi: 10.1136/annrheumdis-2021-220626 PMC8350975 34362747

[46] Bonelli MM Mrak D Perkmann T Haslacher H Aletaha D. SARS-CoV-2 Vaccination in Rituximab-Treated Patients: Evidence for Impaired Humoral But Inducible Cellular Immune Response. Ann Rheum Dis (2021) 80:1355–6. doi: 10.1136/annrheumdis-2021-220408 33958323

[47] Madelon N Lauper K Breville G Sabater Royo I Goldstein R Andrey DO. Robust T Cell Responses in Anti-CD20 Treated Patients Following COVID-19 Vaccination: A Prospective Cohort Study. Clin Infect Dis (2021) ciab954. doi: 10.1093/cid/ciab954 34791081 PMC8767893

[48] Benucci M Damiani A Infantino M Manfredi M Grossi V Lari B. Presence of Specific T Cell Response After SARS-CoV-2 Vaccination in Rheumatoid Arthritis Patients Receiving Rituximab. Immunol Res (2021) 69:309–11. doi: 10.1007/s12026-021-09212-5 PMC8319896 34324159

[49] Bitoun S Henry J Desjardins D Vauloup-Fellous C Dib N Belkhir R. Rituximab Impairs B-Cell Response But Not T-Cell Response to COVID -19 Vaccine in Auto-Immune Diseases. Arthritis Rheumatol (2021) 10.1002/art.42058. doi: 10.1002/art.42058 34962357 PMC9011892

[50] Stumpf J Siepmann T Lindner T Karger C Schwöbel J Anders L. Humoral and Cellular Immunity to SARS-CoV-2 Vaccination in Renal Transplant Versus Dialysis Patients: A Prospective, Multicenter Observational Study Using mRNA-1273 or BNT162b2 mRNA Vaccine. Lancet Reg Heal - Eur (2021) 9:100178. doi: 10.1016/j.lanepe.2021.100178 PMC8299287 34318288

[51] Connolly CM Chiang TP-Y Boyarsky BJ Ruddy JA Teles M Alejo JL. Temporary Hold of Mycophenolate Augments Humoral Response to SARS-CoV-2 Vaccination in Patients With Rheumatic and Musculoskeletal Diseases: A Case Series. Ann Rheum Dis (2022) 81(2):293–5. doi: 10.1136/annrheumdis-2021-221252 PMC11034709 34556484

[52] Curtis JR Johnson SR Anthony DD Arasaratnam RJ Baden LR Bass AR. American College of Rheumatology Guidance for COVID-19 Vaccination in Patients With Rheumatic and Musculoskeletal Diseases: Version 3. Arthritis Rheumatol (2021) 73:1–4. doi: 10.1002/art.41928 PMC8426685 34346564

