# Supplementary material for: Serological Response to BNT162b2 Anti-SARS-CoV-2 Vaccination in Patients with Inflammatory Rheumatic Diseases: Results From the RHEUVAX Cohort
Source: Front Immunol. 2022 Jun 17;13:901055. doi: 10.3389/fimmu.2022.901055 (PMC9247185; doi:10.3389/fimmu.2022.901055)
Supplement: Supplementary file 1 [file Table_1.docx]

1. **Supplementary material**

**Supplementary Table 1 Immunogenicity of BNT162B2 in IRD**

|  | **Seropositivity rate (% of total)** | | **Ln(OR) of non-response (****95% CI)** |
| --- | --- | --- | --- |
|  | **Controls** | **IRD** |  |
|  | *231/232(99.6)* | *210/237 (88.6*)*^a^* | *1.61-5.73* |
| **Age** |  |  |  |
| - *< 59 yrs* | *117/118 (99.2)* | *110/121 (90.9)^a^* | *1.40-5.52* |
| - *> 59 yrs* | *114/114 (100)* | *100/116 (86.2)^a^* | *1.82-5.97* |
| **Sex** |  |  |  |
| - *F* | *160/161 (99.4)* | *144/164 (87.8)^a^* | *1.97-6.05* |
| - *M* | *71/71 (100)* | *66/73 (90.4)^a^* | *1.39-5.62* |
| **IRD** |  | *210/237 (88.6*)*^a^* | *1.61-5.73* |
| - AI | *-* | *4/4 (100)* | *-11-6.26* |
| - CTD | *-* | *52/62 (83.9)^a^* | *1.98-6.18* |
| - RA | *-* | *72/86 (83.7)^a^* | *1.96-6.17* |
| - SpA | *-* | *70/71 (98.6)* | *-1.76-4.14* |
| - Vsc | *-* | *12/14 (85.7)**^a^* | *1.40-6.32* |
| **Treatment - csDMARDs** | *-* | *132/157(84.0)^a^*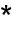 | *2.09-6.12* |
| - *none* | *-* | *78/80 (97.5)* | *-0.39-4.46* |
| - *MTX* | *-* | *82/95 (86.3)^a^**^,b^* | *1.74-5.97* |
| - *HCQ* | *-* | *14/15 (93.3)* | *-0.21-5.74* |
| - *MMF* | *-* | *7/16 (43.8)^a,b^* | *3.73-8.08* |
| - *AZA* | *-* | *16/17 (94.1)* | *-11-5.39* |
| - *SSZ* | *-* | *10/10 (100)* | *-11-5.39* |
| - *LEF* | *-* | *1/2(50)^a,b^* | *1.83-8.37* |
| - *Colchicine* | *-* | *3/3 (100)* | *-11-6.95* |
| - *IVIG* | *-* | *2/2 (100)* |  |
| **Treatment -CCS** |  |  |  |
| - no | *-* | *155/165 (93.9)**^a^* | *0.93-5.09* |
| - yes | *-* | *55/72 (76.4)^a,b^* | *2.46-6.62* |
| **Treatment - bDMARDs** | - | 105/119 (88.24)*^a^* | 1.60-5.79 |
| - none | *-* | *105/118 (89.0)^a^* | *1.51-5.73* |
| - TNFi | *-* | *46/49 (93.9)^a^* | *0.78-5.28* |
| - ABA | *-* | *8/15 (53.3)^a,b^* | *3.29-7.73* |
| - UST | *-* | *7/7 (100)* | *-11-5.70* |
| - IL-17 | *-* | *14/14 (100)* | *-11-5.01* |
| - IL-6 | *-* | *11/12 (91.7)* | *0.02-5.99* |
| - IL-1 | *-* | *2/2 (100)* | *-11-6.95* |
| - JAKi | *-* | *16/18 (88.9)^a^* | *1.13-6.04* |
| - PDE4i | *-* | *1/1 (100)* | *na* |
| - BEL | *-* | *0/1 (0)**^a^* | *na* |
| **Treatment - RTX** |  |  |  |
| - None or >12 months | *-* | *207/232 (89.2)^a^* | *1.54-5.57* |
| - <12 months | *-* | *3/5 (60)^a^* | *2.15-7.19* |
| **Past - COVID-19** |  |  |  |
| - no | *na* | *197/224 (87.9)**^a^* | *1.63-5.79* |
| - yes | *na* | *13/13 (100)* | *-11-5.08* |

^a^ statististically different from CTRL, ^b^ statistically different from IRD not receiving the treatment
